# Novel Radiographic Measurements for Operatively Treated Haglund’s Deformity

**DOI:** 10.3390/tomography8010023

**Published:** 2022-02-01

**Authors:** Shih-Chieh Tang, Kao-Chang Tu, Wei-Jen Liao, Chang-Te Hsu, Han-Ting Shih, Kuan-Kai Tung, Min-Huan Wu, Shun-Ping Wang

**Affiliations:** 1 Department of Orthopaedics, Taichung Veterans General Hospital, Taichung 40705, Taiwan; tom0857tom0857@gmail.com (S.-C.T.); shark310751@gmail.com (K.-C.T.); cavaliarjames@gmail.com (W.-J.L.); h901920@gmail.com (H.-T.S.); david02200918@gmail.com (K.-K.T.); 2 Department of Orthopaedics, Changhua Christian Hospital, Changhua 50006, Taiwan; m1040040@gmail.com; 3 Sports Recreation and Health Management Continuing Studies-Bachelor’s Degree Completion Program, Tunghai University, Taichung 40704, Taiwan; mhwu@thu.edu.tw; 4 Bachelor of Science in Senior Wellness and Sport Science, Tunghai University, Taichung 40704, Taiwan; 5 Department of Post-Baccalaureate Medicine, College of Medicine, National Chung Hsing University, Taichung 40227, Taiwan

**Keywords:** Haglund’s syndrome, heel pain, Haglund’s deformity, pump bump, bump height, bump-calcaneus ratio, Achilles tendon

## Abstract

Background: Haglund’s deformity, which is characterized by a bony prominence of the posterosuperior aspect of the calcaneus, causes posterior heel pain. To date, there is no standard radiographic parameter to diagnose symptomatic Haglund’s deformity. Herein, we proposed novel radiographic measurements to distinguish between patients with and without symptomatic Haglund’s deformity. Methods: We retrospectively evaluated ankle radiographs of 43 patients who underwent surgery for symptomatic Haglund’s deformity (Haglund group) and 41 healthy individuals (control group) free of heel complaints. Fowler–Phillip angle (FPA), Heneghan–Pavlov parallel pitch lines (PPL), Haglund’s deformity height, bump height, and bump-calcaneus ratio were measured and compared between the groups. Furthermore, the reliability and cut-off value of each parameter were validated via ICC and ROC curve analysis, respectively. Results: The bump height (*p* < 0.001) and the bump-calcaneus ratio (*p* < 0.001) showed significant differences between the control and Haglund groups, unlike FPA, PPL, and Haglund’s deformity height. ROC curve analysis revealed that the AUC of bump-calcaneus ratio was larger than that of bump height. The optimal threshold was 4 mm or higher for bump height and 7.5% or higher for bump-calcaneus ratio. The intra- and inter- observer ICCs were, respectively, 0.965 and 0.898 for bump height and 0.930 and 0.889 for bump-calcaneus ratio. Conclusions: This study proposes two novel radiographic parameters to identify operatively treated Haglund’s deformity, namely bump height and bump-calcaneus ratio. They are easy to measure and intuitive. Both of them are effective diagnostic parameters for Haglund’s deformity. Furthermore, bump-calcaneus ratio is more reliable diagnostic parameter than bump height.

## 1. Introduction

Haglund’s deformity, also known as “pump bump,” is one of the common causes of heel pain. It was first described by Patrick Haglund in 1928 [1]. It is characterized by a bony prominence of the posterosuperior aspect of the calcaneus [2,3]. Increased irritation in the surrounding soft tissue, secondary to this bony exostosis, contributes to typical heel pain.

At present, the etiology of Haglund’s deformity is not well understood and may be related to tightness of the Achilles tendon, talipes cavus, heredity and calcaneal morphology or position. Haglund’s deformity usually occurs in middle-aged women and is usually bilateral. It is mainly manifested by swelling and pain in the posterior heels. Conservative treatments, including anti-inflammatory drugs, physiotherapy, orthosis, and adjustment of insole height, constitute the first-line treatment; however, their success rate is low [4]. In general, surgeries are considered if conservative treatment fails. According to previous studies, approximately 50% to 65% of cases with Haglund’s deformity require surgical interventions [5,6]. 

Many studies have proposed various X-ray measurement methods to objectively diagnose and define Haglund’s deformity since its first description in 1928 [7,8,9,10,11]. Conventional X-ray measurement methods, namely Fowler–Phillip angle (FPA), Heneghan–Pavlov parallel pitch lines (PPL) and Chauveaux–Liet angle are commonly used in clinical practice; however, they cannot successfully distinguish between Haglund’s and non-Haglund’s deformities [8,9,10,12]. In addition, Kang et al. used Haglund’s deformity height, an intuitive parameter of Haglund’s deformity, to indicate Haglund’s deformity but they did not determine the validity of this parameter in their research [13]. To date, there are still not effective and worldwide accepted radiographic parameters to distinguish the patient who have symptomatic Haglund’s deformity or not.

This study aimed to validate two new radiographic measurements for the diagnosis of symptomatic Haglund’s deformity and verify their cut-off values and reliability. We hypothesized that our new radiographic measurements would show significant differences between patients with and without operatively treated Haglund’s deformity. The results of this study could provide clinicians with an objective measurement for the diagnosis of Haglund’s deformity and help to establish a consensus among various specialties concerning this issue.

## 2. Materials and Methods

### 2.1. Participant Enrollment

We retrospectively evaluated the lateral view of ankle radiographs of 43 patients who underwent surgeries for Haglund’s deformity (Haglund group) from August 2011 to December 2020 and enrolled the other 41 relatively healthy individuals without posterior heel pain as the control group. The radiographic measurements for Haglund’s deformity were performed in a total of 84 heels. This study was approved by the Institutional Review Board (IRB) of our institution (approval no.: CE21051A).

The Haglund group consisted of 22 males and 21 females with an average age of 56 ± 13.5 years (range, 25–75 years). The clinical diagnostic criteria for Haglund’s deformity included palpable calcaneal hump with posterior heel pain and local swelling in the prominence of the posterior calcaneus. After conservative treatment failed, all patients received MRI to exclude any other pathogenesis or tumor over the posterior heel [14] and underwent calcaneoplasty with open retrocalcaneal decompression performed by one single foot and ankle surgeon in our hospital.

The control group consisted of 19 males and 22 females with an average age of 55 ± 10.8 years (range, 33–72 years). According to their medical records, they had no posterior heel pain, and most of them sustained ankle sprain. 

Exclusion criteria were the same in both the groups: individual with less than 18 years of age, infection in the foot and ankle, inflammatory disease, any previous ankle or foot surgeries, or incomplete medical records.

### 2.2. Radiographic Measurement

To quantify the bone anomalies potentially implicated in posterior heel pain, we used the standard radiologic criteria, and all radiographic parameters were measured on the lateral view radiograph of the ankle using the software built in picture archiving and communications system (PACS) with ultraquery (Taiwan Electronic Data Processing, Sindian City, Taiwan). To decrease the possible variation of ankle radiography by foot malpositioning, the standardized radiographic procedure was followed. The patient was requested to be in a lateral recumbent position on the table and an image receptor was placed vertically beneath the upright ankle with foot in dorsiflexion. The lateral aspect of the knee and ankle joint should closely contact with the table resulting in the tibia lying parallel to the table. The x-ray beam was directed horizontally, centered at the bony prominence of the medial malleolus of the distal tibia. The position of distal fibula, distal tibia and talar domes were checked to evaluate the quality of radiographs. The distal fibula should be superimposed by the posterior portion of the distal tibia and the superior articular surface of the talus should be clearly identified.

**Figure 1:**
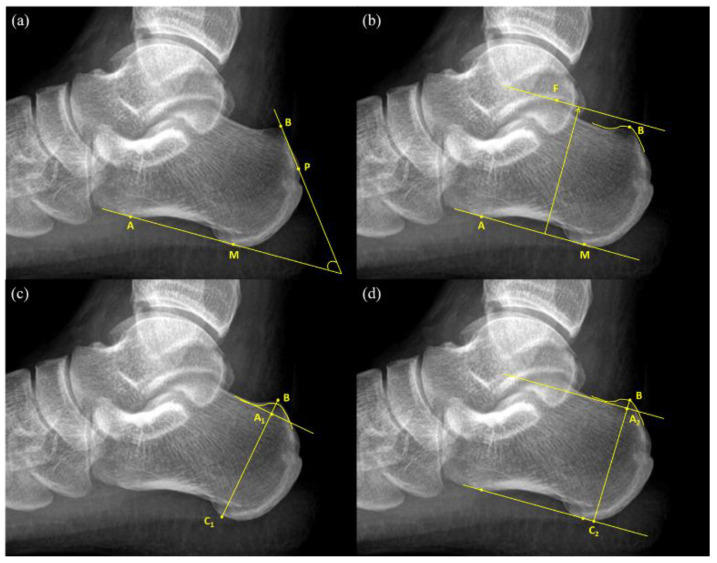
Radiographic measurement of Haglund’s deformity (**a**) Fowler-Philip angle = Angle between $\bar{\mathrm{AM}\;}$ and $\bar{\mathrm{BP}\;};$ (**b**) Heneghan-Pavlov parallel pitch lines; (**c**) Haglund’s height = $\bar{{\mathrm{BA}}_{1}}$; Haglund’s height ratio = $\bar{{\mathrm{BA}}_{1}}/\bar{{\mathrm{BC}}_{1}}$; (**d**) Bump height = $\bar{{\mathrm{BA}}_{2}}$; bump-calcaneus ratio = $\bar{{\mathrm{BA}}_{2}}/\bar{{\mathrm{BC}}_{2}}$.

An FPA is the angle between the tangent line to the rear edge of the greater tuberosity of the calcaneal and the line connecting the anterior tubercle and medial tuberosity of the calcaneal (Figure 1a). The normal values of FPA range from 44° to 69°, and values greater than 75° are considered indicators of pathological changes [9].

Heneghan–Pavlov PPL is the baseline formed by the line connecting the anterior tubercle and medial tuberosity of the calcaneus and a parallel superior line passing through the superior aspect of the talar articulation (Figure 1b). If PPL passes through the prominence of the posterosuperior edge of the calcaneal tuberosity, it is considered positive [10].

Haglund’s deformity height ($\bar{{\mathrm{BA}}_{1}}$) (Figure 1c) was obtained first by drawing a reference line at the base of the posterosuperior calcaneal prominence and then by measuring the vertical distance between the vertex of the bump and the reference line [13]. Next, calcaneal height ($\bar{{\mathrm{BC}}_{1}}$) was measured as the vertical distance between the vertex of the bump and the reference line drawn tangent to the lower edge of the calcaneus. Haglund’s height ratio was calculated as the ratio of Haglund’s deformity height to calcaneal height ($\bar{{\mathrm{BA}}_{1}}/\bar{{\mathrm{BC}}_{1}}$) (Figure 1c).

This study put forward two novel measurement methods for the diagnosis of Haglund’s deformity, namely bump height and bump-calcaneus ratio. Firstly, the baseline tangent to the anterior tubercle and medial tuberosity of the calcaneus was drawn. The baseline was moved upward parallelly until it touched the superior edge of the calcaneus, which was considered the reference line for bump height different to that in Haglund’s deformity height. Then, the reference line was moved upward parallelly again until it reached the superior edge of the bump and the intersection point was considered as the vertex of the bump (point B). The vertical distance between the vertex of the bump and the reference line was measured as bump height ($\bar{{\mathrm{BA}}_{2}}$), whereas the vertical distance between the vertex of the bump and the baseline was measured as calcaneal thickness ($\bar{{\mathrm{BC}}_{2}}$). Then, bump-calcaneus ratio was calculated by dividing bump height ($\bar{{\mathrm{BA}}_{2}}$) by calcaneal thickness ($\bar{{\mathrm{BC}}_{2}}$) (Figure 1d).

In order to assess interobserver reliability, two orthopedic surgeons blindly carried out all measurements using the above-mentioned methods. Additionally, the first author and another orthopedic surgeon conducted the same radiographic measurements. In order to evaluate intraobserver reliability, two observers conducted two identical measurements at an interval of seven days. Each measurement was carried out independently by two observers; it was confirmed that the measurements by two observers would not interfere with each other.

### 2.3. Statistical Analysis

Continuous data including patients’ age are presented as means and standard deviations. The normality of continuous data was checked using the Kolmogorov—Smirnov test. The Mann–Whitney U test was performed to examine intergroup differences in age. Descriptive data, such as patients’ gender and surgical site, are presented as frequencies and percentages. The chi-square analysis and Fisher’s exact test were conducted to compare categorical data between the two groups. The area under the curve (AUC) and optimal cut-off value were calculated from the receiver operating characteristic (ROC) curve. The intraclass correlation coefficient (ICC) and kappa index were used to validate the intraobserver and interobserver reliability of each parameter. All statistical analyses were performed using SPSS version 22.0 (IBM, New York, NY, USA). The level of statistical significance was set at *p* < 0.050. The post hoc power of the two new target parameters was calculated using G*Power 3.1.9.7 (Heinrich-Heine-Universität Düsseldorf, Düsseldorf, Germany) with the setting of α = 0.05.

## 3. Results

### 3.1. Participant Demographics

A total of 84 patients, including 40 males and 44 females, with an average age of 54 years were enrolled in this study. Total 50 right feet and 34 left feet were studied. Among these 84 patients, 43 patients in the Haglund group had symptomatic Haglund’s deformity and underwent surgery in our hospital, whereas 41 patients in the control group had no posterior heel pain. There was no statistically significant difference in age, gender, and laterality between the control and Haglund groups (Table 1).

### 3.2. X-ray Parameters for the Diagnosis of Haglund’s Deformity

There was no statistically significant difference in mean FPA between the Haglund and control groups (62.80 ± 5.66° vs. 61.41 ± 5.43°, *p* = 0.543). No one in our cohort had an FPA greater than 75°, previously reported cut-off value of FPA. There were 19 cases (67.44%) in the Haglund group and 29 cases (46.34%) in the control group with positive PPLs; there was no statistically significant difference between the two groups (*p* = 0.083) (Table 2).

There was no statistically significant difference in Haglund’s deformity height between the Haglund and control groups (7.20 ± 1.29 mm vs. 6.80 ± 0.87 mm, *p* = 0.202). There was also no statistically significant difference in Haglund’s height ratio between the Haglund and control groups (14.51 ± 2.55% vs. 15.04 ± 1.92%, *p* = 0.876) (Table 2).

The Haglund group showed significantly higher average bump height than the control group (4.60 ± 1.35 mm vs. 3.20 ± 1.20 mm, *p* < 0.001). The bump-calcaneus ratio in the Haglund group was significantly higher than that in the control group (9.41 ± 2.26% vs. 6.58 ± 2.13%, *p* < 0.001) (Table 2).

### 3.3. ROC Curve and Cut-Off Value

**Figure 2:**
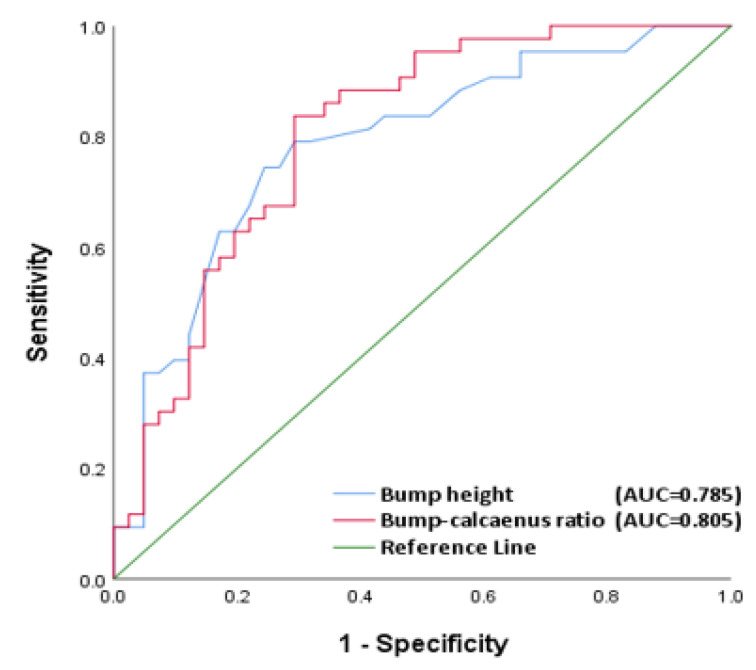
Receiver operating characteristic (ROC) curve analysis for radiographic parameters. The blue curve represents bump height and the red curve represents bump-calcaneus ratio. The AUC of bump height and bump-calcaneus ratio were 0.785 and 0.805, respectively.

According to ROC curve analysis (Figure 2), the AUC of bump height and bump-calcaneus ratio were 0.785 and 0.805, respectively, indicating an acceptable discrimination in bump height and an excellent discrimination in bump-calcaneus ratio (Table 3).

Further ROC curve analysis revealed that the optimal cut-off value of bump height was 4 mm. In the Haglund group, 74.42% (32 of 43) patients had a bump height of 4 mm or more; in the control group, 75.61% (31 of 41) patients had a bump height of less than 4 mm. The sensitivity, specificity, positive predictive value (PPV), and negative predictive value (NPV) of bump height were 74.4%, 75.6%, 76.2%, and 73.8%, respectively (Table 3).

The optimal cut-off value of bump-calcaneus ratio was 7.5%. In the Haglund group, 83.7% (36 of 43) patients had a bump-calcaneus ratio of greater than 7.5%; in the control group, 70.7% (28 of 41) patients had a bump calcaneus ratio of less than 7.5%. The sensitivity, specificity, PPV, and NPV of bump-calcaneus ratio were 83.7%, 68.3%, 73.5%, and 80.0%, respectively (Table 3).

### 3.4. Intraclass Correlation Coefficient (ICC) and Kappa Index

Bump height and bump-calcaneal ratio showed almost a perfect intraobserver agreement (ICC > 0.9) and substantial interobserver agreement (kappa index > 0.8). However, other radiographic parameters showed poor to fair interobserver agreement (Table 4).

## 4. Discussion

This study put forward two novel and highly reproducible measurement methods for the diagnosis of operatively treated Haglund’s deformity, namely bump height and bump-calcaneus ratio. They are also the first continuous variables measured based on the bump height in Haglund’s deformity. Furthermore, considering the influences of body sizes on calcaneal height, we also proposed using the bump-calcaneus ratio. Our results revealed that previous X-ray measurements including FPA, PPL, Haglund’s deformity height, and Haglund’s height ratio could not effectively diagnose symptomatic Haglund’s deformity, whereas bump height and bump-calcaneus ratio showed statistically significant differences between Haglund and non-Haglund groups. Among all the radiographic parameters measured in this study, bump-calcaneus ratio had the highest AUC and quite high reproducibility and accuracy. The intra and interobserver reliability of bump-calcaneus ratio were also excellent, and the sensitivity, specificity, and accuracy of bump-calcaneus ratio were 83.7%, 68.3%, and 76.2%, respectively. The statistical power of the two new target parameters was calculated and reached 0.99.

X-ray measurement methods that are frequently used for the diagnosis of Haglund’s deformity include FPA and PPLs; however, they have poor sensitivity and specificity for the diagnosis of Haglund’s deformity. In 1945, FPA was first put forth by Fowler et al. [9]. Since then, many studies have considered it as the diagnostic standard for Haglund’s deformity. The normal value of FPA ranges from 44° to 69°, and an FPA of more than 75° is regarded as the cut-off value for the diagnosis of Haglund’s deformity [9]. In 2007, Lu et al. first reported that FPA showed no statistically significant difference between the disease and control groups, and subsequent studies supported this conclusion [11,12]. In addition, Fowler et al. did not clarify the basis of using 75° as the diagnostic threshold, and many subsequent studies have shown that even the symptomatic Haglund group rarely had an FPA over 75°9. In our cohort, the mean FPA in the Haglund and control groups were 61.2° and 61.7°, respectively; however, there was no statistically significant difference between the groups. None of this cohort had an FPA greater than 75°. Our findings are compatible with the conclusions of previous studies. In 1982, Pavlov et al. put forward PPLs; it is a measurement method comprising categorical variables and the only one divided dichotomically into pathological and no pathological changes [10]. They reported that PPLs successfully diagnosed Haglund’s deformity by showing a statistically significant difference between the disease and control groups. However, there were only 10 heels in the disease group and 78 heels in the control group. This large difference in sample size between the two groups might have compromised the statistical power, probably leading to inaccurate conclusions. A subsequent study confirmed that there was no significant difference in PPLs between the Haglund group and control group [12]. In this study, we also found no statistically significant difference in PPLs between the Haglund and control groups. Importantly, the previous studies found that FPA and PPLs could not effectively diagnose symptomatic Haglund’s deformity; furthermore, it did not propose a new radiographic measurement method to diagnose this disease.

To the best of our knowledge, scarce radiographic measurement methods with good sensitivity and specificity have been reported for diagnosing symptomatic Haglund’s deformity. Bulstra et al. proposed the calcaneal pitch angle (CPA), defined as the angle between the ground and plantar surface of the calcaneus, could effectively distinguish the Haglund’s group and control group. However, based on their subgroup analysis, the significant difference of CPA was mainly found between female patients but not males in the two group. The gender-specific radiographic parameter may limit its clinical application [15]. Furthermore, Tourné et. al. indicated no significant difference in the CPA between groups and suggested the calcaneal morphology. X/Y ratio, measured as the ratio of the calcaneal length to greater tuberosity length, is a better radiographic measurement to distinguish the Haglund syndrome with very high sensitivity (100%) and specificity (95%). X/Y ratio focused on calcaneus length and offered an objective reference for Zadek osteotomy to treat Haglund syndrome [16]. However, Haglund syndrome is different from Haglund’s deformity. The radiographic parameters applied to Haglund syndrome need to be further validated to distinguish Haglund’s deformity from non-Haglund’s group.

Haglund’s deformity is characterized by a bony prominence of the posterior-superior aspect of the calcaneus. In 2012, Kang et al. used Haglund’s deformity height to verify the relationship between Haglund’s deformity and insertional Achilles tendinitis [13]. They showed that Haglund’s deformity was not directly related to insertion Achilles tendinitis; however, they directly used this parameter to indicate Haglund’s deformity, without verifying its efficacy. In this study, we validated the efficacy and accuracy of Haglund’s deformity height and considered the influence of body size on calcaneal height. Haglund’s height ratio was modified as a new measurement parameter for the diagnose Haglund’s deformity. However, the measurements of both Haglund’s deformity height and Haglund’s height ratio did not easily and clearly define the reference line of the bump because the margin at the base of the bump cannot be visibly identified in some cases.

Therefore, we measured bump height using the connecting line between the anterior tubercle and medial tuberosity on the inferior margin of the calcaneus instead, which is used as the baseline for both FPA measurement and the Heneghan–Pavlov PPLs test. Next, we took into account the effect of body size on calcaneal height. The bump height was divided by the measured calcaneal height ($\bar{{\mathrm{BC}}_{2}}$, in Figure 1d) to yield another new parameter, named bump-calcaneus ratio, to diagnose Haglund’s deformity. Instead of using the reference line directly connecting the margin at the base of calcaneal bump, the reference line used in bump height (ratio) is definite due to obvious bony landmarks in calcaneus. Although Haglund’s deformity height (ratio) and bump height (ratio) have similarly attempted to measure the height of calcaneal bump, the bump height (ratio) has the advantage of distinct reference line at the bump base. This modification of reference line significantly enhanced the reliability and reproducibility. The ICCs correlation was poor in Haglund’s deformity height (ratio) but good to excellent in bump height (ratio).

The existing measurement methods of Haglund’s deformity mostly focus on the changes in calcaneal shape; among them, only PPL, a categorical variable, is related to the bump. The connecting line between the anterior tubercle and medial tuberosity of the calcaneal, which is a well-known feature used in measuring PPL, was used as the baseline for calculating bump height in this study. Next, the bump-calcaneus ratio was calculated by dividing bump height by calcaneus height. Our newly proposed measurements take into account the characteristics of Haglund’s deformity, including bony prominence of the calcaneus and the difference in calcaneus height as per individual body size.

To date, the commonly used measurement methods of Haglund’s deformity cannot determine whether a patient requires operative treatment. All patients in the Haglund group underwent operation after conservative treatment failed. In this study, the new measurement methods based on the lateral view of the ankle revealed that 74.4% of the patients showed symptomatic Haglund’s deformity with a bump height of 4 mm or higher, whereas about 83.7% of the patients showed a bump-calcaneus ratio of 7.5% or larger. At present, the main operative management of Haglund’s deformity is a retrocalcaneal decompression for bump removal or a closing wedge calcaneal osteotomy for changing calcaneus shape [2,17,18,19,20,21]. However, the extent of bump removal is still controversial, and only a few studies have reported this. Sella et al. suggested that the bump removal should be targeted to maintain an FPA between 48° and 49° [20]. In our study, the lower the bump was, the less the clinical symptom was. Therefore, we believe that bump decompression is necessary in patients with symptomatic Haglund’s deformity who required surgery. Surgeons may use a bump height of 4 mm and a bump-calcaneus ratio of 7.5% as references while deciding bump removal height. Unlike Heneghan–Pavlov PPL, which is a category variable, other radiographic parameters are unable to indicate the severity of Haglund’s deformity and cannot provide an operative guidance. Continuous variables, such as bump-calcaneus ratio, may help orthopedic surgeons to predict disease severity and to decide bump decompression volume; however, further studies with a large sample size are needed to confirm efficacy of the parameter.

This study has some limitations, including small sample size and retrospective design, which might lead to selection or undetectable bias. In addition, this study was conducted at a single institution and the control group in our cohort consisted of relatively healthy patients. In the future, multi-center prospective controlled studies including completely healthy patients as the control group are required to verify the accuracy of our measurement methods.

## 5. Conclusions

In this study, we proposed two novel radiographic parameters for the diagnosis of operatively treated Haglund’s deformity, namely bump height and bump-calcaneus ratio. They can both effectively diagnose symptomatic Haglund’s deformity and show good reliability. The cut-off values of bump height and bump-calcaneus ratio are 4 mm and 7.5%, respectively. Our proposed measurements herein may provide clinicians with a guidance for the diagnosis of Haglund’s deformity and for the extent of bump removal during retrocalcaneal decompression.

## Figures and Tables

**Table 1:** Characteristics of the study cases.

	Total (*n* = 84)	Control (*n* = 41)	Haglund (*n* = 43)	*p* Value
Age	54.0 ± 12.3	53.1 ± 10.8	54.8 ± 13.6	0.382 *^a^*
Gender, *n* (%)				0.992 *^b^*
Female	44 (52.38%)	22 (53.66%)	22 (51.16%)	
Male	40 (47.62%)	19 (46.34%)	21(48.84%)	
Side, *n* (%)				0.687 *^b^*
Right	50 (59.52%)	23 (56.10%)	27 (62.79%)	
Left	34 (40.48%)	18 (43.90%)	16 (37.21%)	

Mann—Whitney U test *^a^*. Chi-square test *^b^*. Continuous data are expressed as mean ± SD. Categorical data are expressed as number (percentage).

**Table 2:** A comparison of X-ray parameters for the diagnosis of Haglund’s deformity between the Haglund and control groups.

	Control	Haglund	*p* Value
	(*n* = 41)	(*n* = 43)	
Fowler-Philip angle (FPA), °	62.80 ± 5.66	61.41 ± 5.43	0.543 *^a^*
Cases with FPA > 75 ^o^ (Pathologic), *n* (%)	0 (0%)	0 (0%)	
Positive parallel pitch lines, *n* (%)	19 (46.34%)	29 (67.44%)	0.083 *^b^*
Haglund’s deformity height, mm	6.80 ± 0.87	7.20 ± 1.29	0.202 *^a^*
Haglund’s height ratio, %	15.04 ± 1.92	14.51 ± 2.55	0.876 *^a^*
Bump height, mm	3.20 ± 1.20	4.60 ± 1.35	<0.001 *^a^* **
Bump-calcaneus ratio, %	6.58 ± 2.13	9.41 ± 2.26	<0.001 *^a^* **

Mann—Whitney U test *^a^*. Chi-square test *^b^*, ** *p* < 0.01. Continuous data are expressed as mean ± SD. Categorical data are expressed as number (percentage).

**Table 3:** The cut-off value and area under the curve (AUC) of bump height and bump calcaneus ratio.

	AUC	Cut-Off Value	Sensitivity	Specificity	PPV	NPV	Accuracy
Bump height	0.785	4	74.4%	75.6%	76.2%	73.8%	75.0%
Bump-calcaneus ratio, %	0.805	7.5	83.7%	68.3%	73.5%	80.0%	76.2%

Positive predictive value (PPV), negative predictive value (NPV).

**Table 4:** Intraobserver and interobserver reliability.

	Inter Observer		Intra Observer	
	Intraclass Correlation	Kappa Index	Spearman’s Rho	Kappa Index
Fowler-Philip angle	0.671		0.646	
Heneghan-Pavlov parallel pitch lines		0.639		0.742
Haglund’s height	0.645		0.655	
Haglund’s height ratio	0.180		0.683	
Bump height	0.898		0.965	
Bump-calcaneus ratio	0.889		0.930	

## Data Availability

All data are available upon reasonable request from the corresponding author.
